# Maternal hypothyroxinaemia in pregnancy is associated with obesity and adverse maternal metabolic parameters

**DOI:** 10.1530/EJE-15-0866

**Published:** 2016-01

**Authors:** Bridget A Knight, Beverley M Shields, Andrew T Hattersley, Bijay Vaidya

**Affiliations:** 1 NIHR Exeter Clinical Research Facility, University of Exeter Medical School, University of Exeter, Exeter, UK; 2 Department of Endocrinology, Royal Devon and Exeter Hospital NHS Foundation Trust, Exeter, EX2 5DW, UK; 3 Research and Development Department, Royal Devon and Exeter Hospital NHS Foundation Trust, Exeter, UK

## Abstract

**Objective:**

Subclinical hypothyroidism and isolated hypothyroxinaemia in pregnancy have been associated with an increased risk of gestational diabetes. We aimed to ascertain if these women have a worse metabolic phenotype than euthyroid pregnant women.

**Design, subjects and methods:**

We recruited 956 healthy Caucasian women with singleton, non-diabetic pregnancies from routine antenatal clinics. Detailed anthropometric measurements (including BMI and skinfold thickness) and fasting blood samples (for TSH, free thyroxine (FT_4_), free triiodothyronine (FT_3_), HbA1c, lipid profile, plasma glucose and insulin resistance (HOMA-IR) analysis) were obtained at 28 weeks gestation.

**Results:**

In comparison to euthyroid women (*n*=741), women with isolated hypothyroxinaemia (*n*=82) had significantly increased BMI (29.5 vs 27.5 kg/m^2^, *P*<0.001), sum of skinfolds (57.5 vs 51.3 mm, *P*=0.002), fasting plasma glucose (4.5 vs 4.3 mmol/l, *P*=0.01), triglycerides (2.3 vs 2.0 mmol/l, *P*<0.001) and HOMA-IR (2.0 vs 1.3, *P*=0.001). Metabolic parameters in women with subclinical hypothyroidism (*n*=133) were similar to those in euthyroid women. Maternal FT_4_ was negatively associated with BMI (*r*=−0.22), HbA1c (*r*=−0.14), triglycerides (*r*=−0.17), HOMA-IR (*r*=−0.15) but not total/HDL cholesterol ratio (*r*=−0.03). Maternal FT_3_:FT_4_ ratio was positively associated with BMI (*r*=0.4), HbA1c (*r*=0.21), triglycerides (*r*=0.2), HOMA–IR (*r*=0.33) and total/HDL cholesterol ratio (*r*=0.07). TSH was not associated with the metabolic parameters assessed.

**Conclusions:**

Isolated hypothyroxinaemia, but not subclinical hypothyroidism, is associated with adverse metabolic phenotype in pregnancy, as is decreasing maternal FT_4_ and increasing FT_3_:FT_4_ ratio. These associations may be a reflection of changes in the thyroid hormone levels secondary to increase in BMI rather than changes in thyroid hormone levels affecting body weight and related metabolic parameters.

## Introduction

Recently, increasing numbers of studies have shown associations between mild maternal thyroid hormone insufficiency (including subclinical hypothyroidism and isolated hypothyroxinaemia) in pregnancy and impaired neuropsychological development of the offspring, as well as several obstetric complications, such as miscarriage, preterm delivery, gestational hypertension and pre-eclampsia (1, 2, 3). Several of these studies (4, 5, 6), although not all (7, 8) have also shown that subclinical hypothyroidism in pregnancy is associated with an increased risk of gestational diabetes. Isolated hypothyroxinaemia has also been shown to be associated with gestational diabetes (7), and a recent study found that lower free thyroxine (FT_4_) levels and higher free triiodothyronine (FT_3_) to FT_4_ ratios (suggesting higher peripheral deiodinase activity leading to conversion of FT_4_ to FT_3_) in pregnant women are associated with several adverse metabolic parameters relating to obesity, glycaemia, insulin resistance and lipid profile (9). It is possible that these adverse metabolic parameters partly explain the poor obstetric outcomes observed in pregnant women with mild maternal thyroid insufficiency (10, 11, 12).

As little is known on whether mild maternal thyroid hormone insufficiency in pregnancy is associated with adverse metabolic parameters, our study aimed to explore the relationship between maternal thyroid hormone levels and metabolic parameters in pregnancy and ascertain if pregnant women with subclinical hypothyroidism and isolated hypothyroxinaemia have worse metabolic phenotype than euthyroid pregnant women.

## Subjects and methods

### Subjects

A total of 988healthy non-diabetic Caucasian women with singleton pregnancies were recruited into the Exeter Family Study of Childhood Health between 1999 and 2004 (13). These women had detailed anthropometric measurements, including height, weight and skinfold measures (triceps, biceps and subscapular) and fasting blood samples taken at 28 weeks gestation. After exclusion of women on thyroid-related medications (levothyroxine *n*=18, propylthiouracil *n*=3) and women with overt biochemical hypothyroidism (*n*=10) or hyperthyroidism (*n*=1) on the blood samples, data from 956 women were included in this study.

The study was approved by the North and East Devon Local Research Ethics Committee, and all participants gave informed written consent.

### Analysis of thyroid function and biochemical metabolic parameters

Thyroid function tests (thyroid-stimulating hormone (TSH), FT_4_ and FT_3_) and thyroid peroxidase antibodies (TPO-Ab) were measured on the stored serum samples. Serum TSH, FT_4_ and FT_3_ were analysed using the electrochemiluminescent immunoassay, run on the Modular E170 Analyzer (Roche). Intra-assay coefficients of variations were as follows: TSH <5.3%, FT_4_ <5.3% and FT_3_ <5.1%. The manufacturer's population reference ranges were as follows: TSH 0.35–4.5 mIU/l, FT_4_ 11–24 pmol/l, and FT_3_ 3.9–6.8 pmol/l*.* High TSH in this study of women in the third trimester was defined as >3 mIU/l, the generally accepted upper limit of the reference range in the second and third trimesters in Caucasian populations (1, 2). Subclinical hypothyroidism was defined as high TSH (>3 mIU/l) with FT_4_ within the reference range (11–24 pmol/l). Isolated maternal hypothyroxinaemia was defined as FT_4_ below the 10th centile (<10.4 pmol/l) with TSH within the reference range. TPO-Abs were analysed using the competitive immunoassay (Roche), and a titre above 34 IU/ml was considered positive.

Fasting plasma glucose, serum insulin and lipid profile and glycosylated haemoglobin (HbA1c) were analysed in the fresh blood samples, as previously described (13). We analysed homeostasis model of assessment for insulin resistance (HOMA-IR) to estimate fasting insulin sensitivity using the following formula: fasting serum insulin (mIU/l)×fasting plasma glucose (mmol/l)/22.5.

### Statistical analysis

We assessed variables for distribution and log-transformed where they were not normally distributed. We used the independent sample *t*-test to compare metabolic parameters in women with isolated hypothyroxinaemia or subclinical hypothyroidism and women with normal thyroid function, and in euthyroid women with and without TPO-Ab. We used the *χ* ^2^ test to assess TPO-Ab-positive proportions in each group. We used Pearson's correlation to assess associations between variables, and multiple linear regression analysis to assess independent associations between variables. As smoking and TPO-Ab are potential confounders affecting thyroid function, we have also included these factors in the regression analysis (14, 15). We used independent samples *t*-test to compare metabolic parameters between women with lower and upper quartiles of FT_4_ and between women with lower and upper quartiles of FT_3_:FT_4_ ratio. We carried out statistical analyses using SPSS (version 22).

## Results

### Study population

Participants had a mean (s.d.) age of 30.1 (5.1) years and BMI of 27.9 (4.6) kg/m^2^; 46% were primiparous and 14% smoked during pregnancy.

### Metabolic parameters in pregnant women with subclinical hypothyroidism and isolated hypothyroxinaemia

A total of 133 (13.9%) women in our cohort had subclinical hypothyroidism and 82 (8.6%) had isolated hypothyroxinaemia.

There was no difference in metabolic parameters between women with subclinical hypothyroidism and euthyroid women (Table 1). Metabolic parameters in women with subclinical hypothyroidism who had TSH greater than 5 mIU/l (*n*=12) were also similar to those in euthyroid women (data not shown).

When compared to euthyroid women, women with maternal isolated hypothyroxinaemia had significantly increased BMI (29.5 vs 27.5 kg/m^2^, *P*<0.001), sum of skinfolds (57.5 vs 51.3 mm, *P*=0.002), fasting plasma glucose (4.5 vs 4.3 mmol/l, *P*=0.01), triglycerides (2.3 vs 2.0 mmol/l, *P*<0.001) and HOMA-IR (2.0 vs 1.3, *P*=0.001), and borderline increased HbA1c (29.9 vs 28.7 mmol/mol, *P*=0.05) (Table 1).

### Association between maternal thyroid hormone levels and metabolic parameters

In bivariate analyses, maternal FT_4_ was negatively associated with BMI (*r*=−0.22, *P*<0.001), sum of skinfolds (*r*=−0.22, *P*<0.001), HbA1c (*r*=−0.14, *P*<0.001), fasting plasma glucose (*r*=−0.13, *P*<0.001), triglycerides (*r*=−0.17, *P*<0.001), HOMA-IR (*r*=−0.15, *P*<0.001) but not Total/HDL cholesterol ratio (*r*=−0.03, *P*=0.3) (Table 2). Maternal FT_3_:FT_4_ ratio was positively associated with BMI (*r*=0.40), sum of skinfolds (*r*=0.37), HbA1c (*r*=0.21), fasting plasma glucose (*r*=0.24), triglycerides (*r*=0.2) and HOMA–IR (*r*=0.33) (*P*<0.001 for all) and Total/HDL cholesterol (*r*=0.07, *P*=0.04). Maternal TSH in pregnancy was not correlated with any of the maternal metabolic parameters assessed.

Women with the lowest FT_4_ quartile and the highest FT_3_:FT_4_ ratio quartile had significantly higher BMI, sum of skinfolds, HbA1c, fasting plasma glucose, triglycerides and HOMA-IR as compared to women with the highest FT_4_ quartile and the lowest FT_3_:FT_4_ ratio quartile respectively (*P*≤0.001 for all) (Table 3). There was no difference in total/HDL cholesterol ratio between groups.

In multivariable regression analyses (Table 4), FT_4_ was independently associated with BMI, HbA1c, triglycerides and smoking during pregnancy, but not HOMA-IR. FT_3_:FT_4_ ratio was independently associated with BMI, HbA1c, HOMA-IR, and borderline associated with smoking during pregnancy, but not triglycerides or total/HDL cholesterol.

### Association between maternal TPO-Ab status and metabolic parameters

Amongst the euthyroid women, there was no difference in metabolic parameters between TPO-Ab-positive (*n*=37) and TPO-Ab (*n*=696)-negative women (data not shown).

## Discussion

In this study of healthy non-diabetic pregnant women, we found that women with isolated hypothyroxinaemia have worse metabolic parameters with increased obesity, glycaemia, triglycerides and insulin resistance compared to euthyroid women. In contrast, subclinical hypothyroidism in pregnancy was not associated with these adverse metabolic parameters. We also identified lower maternal serum FT_4_ and higher FT_3_:FT_4_ ratio (indicating an increased peripheral deiodinase activity leading to conversion of FT_4_ to FT_3_) associated with adverse metabolic parameters.

Thyroid hormones play key roles in regulating metabolic processes and energy homeostasis in the body (16). It is well known that overt thyroid dysfunction (hypothyroidism or hyperthyroidism) affects body weight, and recent studies have shown that even small variations in thyroid hormone levels within the reference range are associated with significant metabolic consequences and changes in body weight (16, 17). In the mouse model of type 2 diabetes, thyroid hormone has been shown to improve glycaemia and insulin sensitivity (18). Our finding of the inverse correlation between maternal serum FT_4_ and maternal BMI is consistent with previous studies in the pregnant and non-pregnant populations (19, 20, 21, 22, 23). In the general population, low FT_4_ as well as high FT_3_:FT_4_ ratio have also been shown to be associated with adverse metabolic parameters, including less favourable lipid profile, blood pressure and insulin resistance (24, 25, 26, 27, 28). It is thought that lower FT_4_ levels are compensated by a higher peripheral deiodinase activity resulting in higher conversion of FT_4_ to active thyroid hormone FT_3_ and higher FT_3_:FT_4_ ratio (20). In a recent study of 321 healthy pregnant women without a history of thyroid dysfunction, Bassols *et al*. (9) found that decreasing FT_4_ and increasing FT_3_:FT_4_ ratios are similarly associated with less favourable metabolic profile. In addition to confirming the associations in a larger cohort of pregnant women, our study also demonstrates the presence of worse metabolic parameters in women with maternal hypothyroxinaemia as compared to euthyroid women. In contrast, despite several studies (4, 5, 6), but not all (7, 8), showing association between subclinical hypothyroidism in pregnancy and gestational diabetes, we were unable to demonstrate that pregnant women with subclinical hypothyroidism have less favourable metabolic parameters than euthyroid pregnant women. The possible explanations for this discrepant observations include differences in study populations (for example, women with gestational diabetes were excluded in our study) and different TSH cut-off levels used to define subclinical hypothyroidism.

As it is a cross-sectional observational study, our study cannot ascertain that the observed less favourable metabolic parameters are caused by changes in the thyroid hormone levels. Indeed, it is possible that the associations between maternal thyroid function and metabolic parameters are mediated by obesity, as glycaemia, insulin resistance and dyslipidaemia are closely associated with obesity. Several lines of evidence support the hypothesis that changes in thyroid hormone levels are the consequence rather than the cause of changes in body weight. Increased subcutaneous fat has been shown to be associated with lower FT_4_ and higher TSH levels in euthyroid adults (29). A study in iodine-deficient pregnant women showed that obesity is associated with an increased risk of maternal hypothyroxinaemia (30). It is thought that obesity stimulates peripheral deiodinase activity as an adaptation process to increase energy expenditure resulting in an increased conversion of FT_4_ to FT_3_ and high FT_3_:FT_4_ ratio (22). More importantly, weight loss following dietary and life-style interventions have been shown to be associated with changes in thyroid hormone levels, including decreases in FT_3_ level and FT_3_:FT_4_ ratio (31 32). Together, these observations suggest that pregnant women should be encouraged to avoid excess weight gain to prevent maternal hypothyroxinaemia.

We acknowledge a number of other limitations in this study. First, we carried out thyroid function tests on stored serum samples; however, it has been shown that serum TSH, FT_4_, FT_3_ and TPO-Ab concentrations remain stable after storage for several years (33). Second, women with gestational diabetes were excluded from the study during recruitment, therefore we were unable to analyse whether the observed association between isolated hypothyroxinaemia and adverse metabolic parameters extended to an increased risk of gestational diabetes. Finally, we were also unable to assess the potential impact of iodine deficiency on our results due to a lack of urinary iodine concentration data.

In conclusion, this study demonstrates that maternal isolated hypothyroxinaemia, but not subclinical hypothyroidism, is associated with adverse metabolic parameters in pregnancy. The association between low serum thyroxine level and adverse metabolic parameters may be a reflection of changes in the thyroid hormone levels secondary to increases in BMI rather than changes in thyroid hormone levels affecting body weight and related metabolic parameters.

## Figures and Tables

**Table 1:** Comparison of thyroid function and metabolic parameters between euthyroid mothers and those with subclinical hypothyroidism and euthyroid mothers and those with isolated hypothyroxinaemia in pregnancy (data presented as mean±s.d. or ^a^geometric mean (s.d. range) except for TPO-Ab positivity which is presented as percentage). The bold values are statistically significant results.

**Metabolic parameters**	**Euthyroid controls** (*n*=741)	**Subclinical hypothyroidism** (*n*=133)	***P* value** ^b^	**Isolated hypothyroxinaemia** (*n*=82)	***P* value** ^b^
TSH^a^ (mIU/l)	1.6 (0.9, 2.7)	3.7 (3.1, 4.5)	**<** **0.001**	1.8 (1.3, 2.5)	**0.001**
FT_4_ (pmol/l)	12.5 (1.4)	11.9 (1.5)	**<** **0.001**	9.7 (0.5)	**<** **0.001**
FT_3_ (pmol/l)	4.2 (0.5)	4.2 (0.5)	0.2	4.1 (0.4)	**0.04**
TPO-Ab positive (%)	5.0	13.7	**0.001**	6.0	0.6
BMI (kg/m^2^)^a^	27.5 (23.4–32.4)	27.5 (23.7–32.1)	0.7	29.5 (25.1–34.7)	**<** **0.001**
Sum of skinfolds (mm)^a^	51.3 (37.2–70.8)	50.1 (33.3–69.2)	0.9	57.5 (40.7–81.3)	**0.002**
HbA1c (mmol/mol)	28.7 (3.7)	28.5 (3.9)	0.7	29.9 (4.6)	0.05
Fasting plasma glucose (mmol/l)	4.3 (0.4)	4.3 (3.8)	0.8	4.5(0.4)	**0.01**
Triglycerides (mmol/l)^a^	2.0 (1.5–2.8)	2.0 (1.4–2.8)	0.7	2.3 (1.7–3.3)	**<** **0.001**
HOMA-IR^a^	1.3 (0.7–2.0)	1.0 (0.8–2.0)	0.4	2.0 (0.9–2.8)	**0.001**
Total/HDL cholesterol ratio	3.4 (0.9)	3.3 (0.9)	0.4	3.5 (1.0)	0.1

a Denotes log-transformed data.

b *P* values refer to euthyroid controls vs subclinical hypothyroidism and euthyroid controls vs isolated hypothyroxinaemia.

**Table 2:** Correlations between maternal TSH, FT_4_, FT_3_:FT_4_ ratio and metabolic parameters at 28 weeks gestation. The bold values are statistically significant results.

**Metabolic parameters**	**TSH** ^a^ *r* (*P* value)	**FT_4_** *r* (*P* value)	**FT_3_:FT_4_ ratio** *r* (*P* value)
BMI (kg/m^2^)^a^	0.007 (0.8)	**−0.22 (** **<** **0.001)**	**0.40** **(** **<** **0.001)**
Sum of skinfolds (mm)^a^	0.013 (0.7)	**−0.22 (** **<** **0.001)**	**0.37 (** **<** **0.001)**
HbA1c (mmol/mol)	0.007 (0.8)	**−0.14 (** **<** **0.001)**	**0.21 (** **<** **0.001)**
Fasting plasma glucose (mmol/l)	−0.01 (0.8)	**−0.13 ** **(** **<** **0.001)**	**0.24 (** **<** **0.001)**
Triglycerides (mmol/l)^a^	0.006 (0.9)	**−0.17 (** **<** **0.001)**	**0.20 (** **<** **0.001)**
HOMA–IR^a^	0.02 (0.5)	**−0.15 (** **<** **0.001)**	**0.33 (** **<** **0.001)**
Total/HDL cholesterol ratio	−0.04 (0.2)	−0.03 (0.3)	**0.07 (0.04)**

a Denotes log-transformed data.

**Table 3:** Comparisons between upper and lower FT_4_ and FT_3_:FT_4_ ratio quartiles, and maternal metabolic parameters (data presented as mean±s.d. or ^a^geometric mean (s.d. range)). Bold values are statistically significant results.

**Metabolic parameters**	**FT_4_**		***P* value**	**FT_3_:FT_4_ ratio**		***P* value**
Lower quartile	Upper quartile	Lower quartile	Upper quartile
BMI (kg/m^2^)^a^	28.8 (24.6–33.9)	26.9 (22.9–31.6)	**<** **0.001**	25.1 (21.9–28.8)	30.2 (26.7–35.5)	**<** **0.001**
Sum of skinfolds (mm)^a^	57.4 (41.7–79.4)	47.9 (34.7–66.1)	**<** **0.001**	44.7 (32.4–61.7)	63.1 (45.7–87.1)	**<** **0.001**
HbA1c (mmol/mol)	29.5 (4.1)	28.1 (3.8)	**0.001**	27.9 (3.6)	29.9 (4.4)	**<** **0.001**
Fasting plasma glucose (mmol/l)	4.4 (0.4)	4.3 (0.4)	**<** **0.001**	4.2 (0.3)	4.5 (0.4)	**<** **0.001**
Triglyceride (mmol/l)^a^	2.2 (1.6–3.1)	1.9 (1.4–2.5)	**<** **0.001**	1.9 (1.4–2.6)	2.2 (1.6–3.1)	**<** **0.001**
HOMA-IR^a^	2.0 (1.0–2.6)	1.0 (0.8–2.0)	**<** **0.001**	1.0 (0.6–1.6)	2.0 (1.0–2.5)	**<** **0.001**
Total/HDL cholesterol ratio	3.0 (1.8)	3.3 (0.7)	0.17	3.3 (0.9)	3.4 (1.0)	0.24

a Log-transformed data.

**Table 4:** Multivariable regression analysis to demonstrate the relationships between maternal FT_4_ and FT_3_:FT_4_ ratio with metabolic parameters. Partial *R* is the adjusted correlation coefficient. Bold values are statistically significant results.

**Associated metabolic parameters**	**Partial *R***	***β***	**95% CI for *β***	***t***	***P***
Maternal FT_4_					
BMI (kg/m^2^)^a^	−0.13	−1.55	−2.43, −0.68	−3.47	**0.001**
HbA1c (mmol/mol)	−0.09	−0.04	−0.07, −0.01	−2.54	**0.01**
Triglycerides (mmol/l)^a^	−0.10	−0.49	−0.84, −0.13	−2.71	**0.007**
HOMA-IR^a^	−0.02	−0.06	−0.31, 0.21	−0.43	0.7
Smoking during pregnancy	0.09	0.43	0.10, 0.77	2.52	**0.01**
TPO-Ab positive	−0.004	−0.02	−0.47, 0.41	−0.10	0.9
Maternal FT_3_:FT_4_ ratio					
BMI (kg/m^2^)^a^	0.26	0.10	0.07, 0.12	7.18	**<** **0.001**
HbA1c (mmol/mol)	0.11	0.001	0.00, 0.002	2.92	**0.004**
Triglycerides (mmol/l)^a^	0.07	0.01	−0.001, 0.02	1.86	0.06
HOMA-IR^a^	0.14	0.02	0.007, 0.023	3.72	**<** **0.001**
Total/HDL cholesterol	−0.07	−0.004	−0.009, 0.000	−3.52	0.06
Smoking during pregnancy	0.07	0. 01	0.00, 0.02	1.98	0.05
TPO-Ab positive	0.02	0.003	−0.01, 0.02	0.80	0.9

a Variable transformed using natural logs (allowing β coefficients to be interpreted in terms of percentage change).
